# Three-terminal RGB full-color OLED pixels for ultrahigh density displays

**DOI:** 10.1038/s41598-018-27976-z

**Published:** 2018-06-26

**Authors:** Markus Fröbel, Felix Fries, Tobias Schwab, Simone Lenk, Karl Leo, Malte C. Gather, Sebastian Reineke

**Affiliations:** 1 Dresden Integrated Center for Applied Physics and Photonic Materials (IAPP) and Institute for Applied Physics, Technische Universität Dresden, Nöthnitzer Str. 61, D-01187 Dresden, Germany; 2 Organic Semiconductor Centre, SUPA, School of Physics and Astronomy, University of St Andrews, North Haugh, St Andrews, KY16 9SS UK

## Abstract

In recent years, the organic light-emitting diode (OLED) technology has been a rapidly evolving field of research, successfully making the transition to commercial applications such as mobile phones and other small portable devices. OLEDs provide efficient generation of light, excellent color quality, and allow for innovative display designs, e.g., curved shapes, mechanically flexible and/or transparent devices. Especially their self emissive nature is a highly desirable feature for display applications. In this work, we demonstrate an approach for full-color OLED pixels that are fabricated by vertical stacking of a red-, green-, and blue-emitting unit. Each unit can be addressed separately which allows for efficient generation of every color that is accessible by superpositioning the spectra of the individual emission units. Here, we use a combination of time division multiplexing and pulse width modulation to achieve efficient color mixing. The presented device design requires only three independently addressable electrodes, simplifying both fabrication and electrical driving. The device is built in a top-emission geometry, which is highly desirable for display fabrication as the pixel can be directly deposited onto back-plane electronics. Despite the top-emission design and the application of three silver layers within the device, there is only a minor color shift even for large viewing angles. The color space spanned by the three emission sub-units exceeds the sRGB space, providing more saturated green/yellow/red colors. Furthermore, the electrical performance of each individual unit is on par with standard single emission unit OLEDs, showing very low leakage currents and achieving brightness levels above 1000 cd/m^2^ at moderate voltages of around 3–4 V.

## Introduction

Displays based on organic light-emitting diodes (OLEDs) are rapidly evolving into serious competitors to the current state-of-the-art liquid crystal display (LCD) technology and can already be found in mobile phones and television screens. OLED based displays provide many advantages, for example, mechanical flexibility which allows the fabrication of displays on curved surfaces. Furthermore, each OLED pixel is self-emissive and does therefore not require illumination by a backlight. As a result, each individual pixel can be switched off completely, thus achieving very high contrast ratios that are superior to those achievable by current LCD technology.

In a typical flat-panel display (LCD or OLED display), each pixel consists of laterally separated red (R), green (G), and blue (B) sub-pixels in a side-by-side geometry. This design suffers from several drawbacks. For example, to display a purely red image, only one third of all sub-pixels are turned on and — assuming all sub-pixels are of the same size — only a third of the active display area, contributes to the overall emission. The requirement to structure each pixel into three sub-pixels also limits the pixel density and achievable resolution. In case of OLED displays, side-by-side layouts are prone to cross contamination during the fabrication process. If, for example, the green-emitting layer contains small amounts of the red emitter, then the green emission will be severely quenched and the nominally green sub-pixel will emit yellow to orange light, depending on the degree of contamination. Furthermore, creating the sub-pixel pattern requires complex and delicate fine metal shadow mask technology or the use of laminated color filters. The latter limits overall device efficiency and the former struggles to provide the accuracy required for the smaller pixel size and higher pixel density of next generation OLED displays.

**Figure 1:**
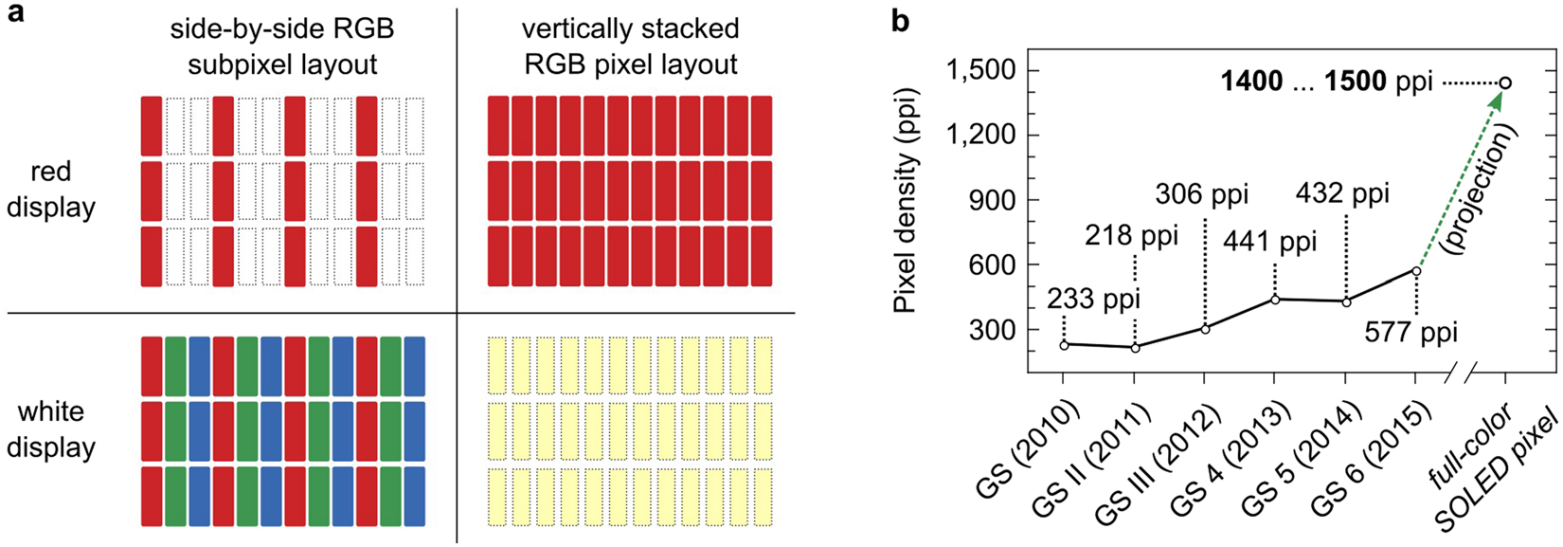
(**a**) Comparison between a standard side-by-side RGB sub-pixel layout and a full-color stacked OLED pixel layout for the two display states “red” and “white”. (**b**) Evolution of display pixel densities for the Samsung Galaxy S smartphone series for the last five years from 2010–2015, as well as an estimate for a future display based on vertical sub-pixel geometry. The pixel densities are given in pixel-per-inch (ppi).

A very promising approach which solves some of these issues in a very elegant way is to use a different pixel geometry in which the R, G, and B sub-pixels are vertically stacked on top of each other. In first approximation, the pixel density and fill factor in this configuration can be up to three times higher than for the conventional side-by-side sub-pixel geometry, as illustrated in Fig. 1a. The stacked RGB configuration is therefore a highly attractive approach to meet the display industry’s demand for ever higher pixel densities as illustrated in Fig. 1.

There are only few publications that describe vertical stacking of three emission units for a full-color OLED pixel. The most important work on this topic has been published almost two decades ago, however, when compared to current state-of-the-art display technology, the devices have only rather low brightness, poor electrical performance, low efficiency, and only mediocre color gamut^1,2^.

In this work, we solve the performance issues of vertically stacked OLEDs with three independently addressable emission units and demonstrate full-color stacked OLED (SOLED) pixels which consist of a red-, green-, and blue-emitting unit. Each unit can be addressed separately which allows to efficiently generate every color within the large color gamut defined by the individual emission units. The resulting color space is then compared to the sRGB color space and it is found that the presented full-color SOLED device not only covers the sRGB color space, but can display more saturated green/greenish-yellow/yellow colors^3^. The device is built in a top-emission geometry which is highly desirable for display fabrication as the pixel can be directly deposited onto the back-plane electronics^4^. Furthermore, our device design requires only three instead of four independently addressable electrodes which simplifies fabrication and electrical driving of these devices. The three emitting units are p-i-n OLEDs, comprising p- and n-doped transport layer systems, separated by highly transparent gold/silver thin-film metal electrodes^5–8^. However, despite the top-emission design and the application of three silver layers within the device, the emission color of each unit is nearly independent of the viewing angle and therefore very well suited for display applications. The electrical performance of each individual unit is on par with conventional p-i-n single emission unit OLEDs, showing very low leakage currents in the range of 10^−3^ mA/cm^2^ and achieving high current densities of 100 mA/cm^2^ at moderate voltages of only 4 V. The latter is an important prerequisite for integration into CMOS back-plane electronics where low pixel voltages are generally desired.

## Results

**Figure 2:**
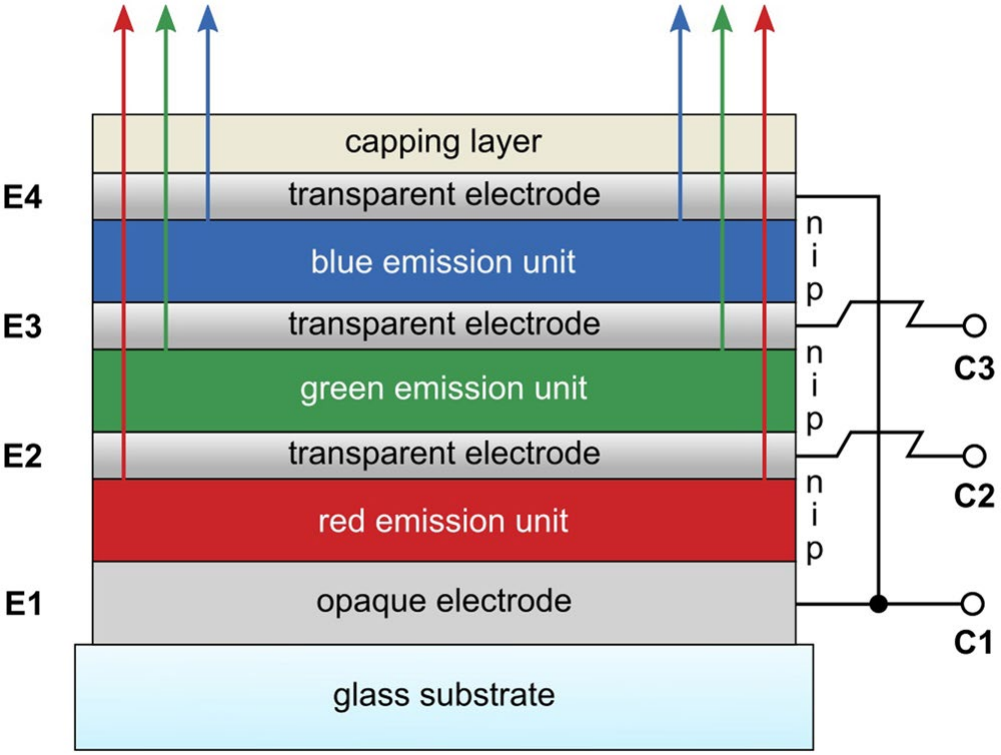
Schematic of the vertically stacked RGB pixel design developed here. E1 to E4 are the internal electrodes, whereas C1 to C3 represent the external terminals which can be accessed to individually drive any of the three emission units. Electrodes E1 and E4 are connected to each other, effectively reducing the amount of required terminals to only three. A detailed schematic of the device architecture, as well as an equivalent circuit diagram is given in Supplementary Fig. 1.

A schematic of the sample architecture is shown in Fig. 2. In the following, we will explain how each sub-unit of our stacked device is operated. The red unit is controlled by putting C1 on a positive potential with respect to C2. In this configuration, the n-side of the blue-emitting unit is connected to a positive potential (by C1) and the p-side of the green unit is on a negative potential (by C2). Contacting the blue and the green unit works analogously. For green emission, C2 must be on a positive potential and C3 on a negative potential. Applying a positive voltage to C3 and a negative voltage to C1 leads to emission from the blue unit. The described addressing scheme allows us to independently investigate the electrical and optical characteristics of the red-, green-, and blue-emitting unit.

**Figure 3:**
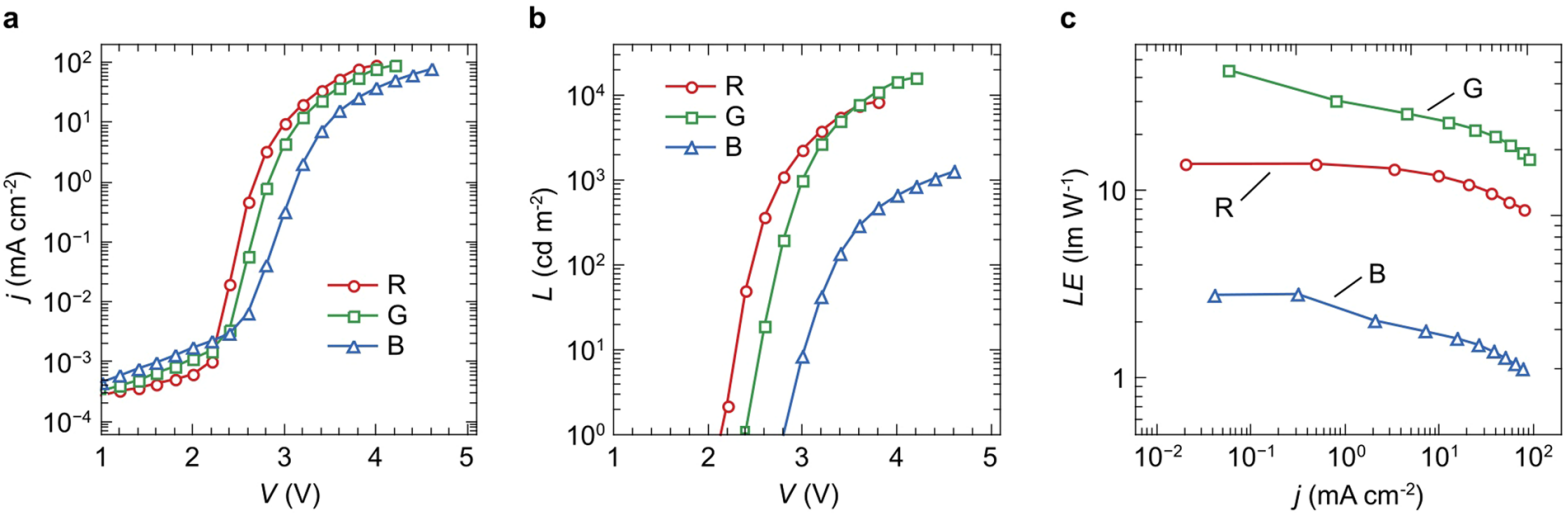
(**a**) Current density (*j*) and (**b**) luminance (*L*) as functions of voltage (*V*). The corresponding luminous efficacy (*LE*) as a function of current density is presented in (**c**).

Figure 3a shows the current density *j* as a function of the applied voltage *V* for each unit. We find that leakage currents are very low, in the range of 10^−4^–10^−3^ mA/cm^2^, and diode onset voltages are below 3 V for all three units. The slope of the *j*-*V* curves is steep and a current density of 100 mA/cm^2^ is already obtained at 4–4.6 V. These results demonstrate that the vertical stacking of three emission units has no adverse effects on the electrical performance of each individual unit.

**Figure 4:**
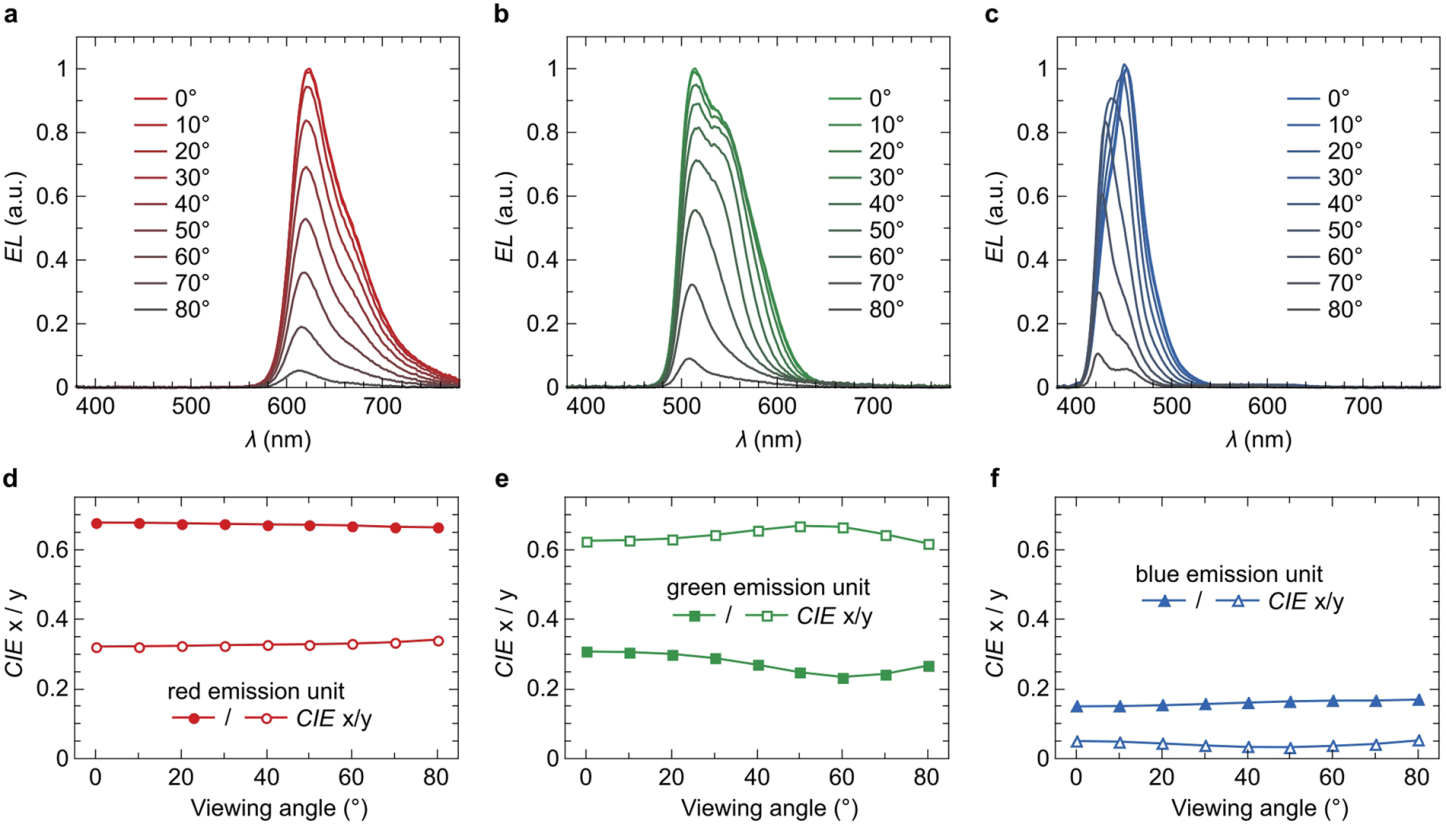
Emission spectra of the (**a**) red, (**b**) green, and (**c**) blue-emitting unit under viewing angles ranging from 0° to 80°. The corresponding CIE color coordinates for the red, green, and blue unit are provided in (**d**), (**e**), and (**f**), respectively.

The light emission onset voltage which is shown in the luminance-voltage (*L*-*V*) characteristics in Fig. 3b is at around 2.2 V for the red-emitting, 2.4 V for the green-emitting, and 2.8 V for the blue-emitting unit. At 4 V, we measure luminance levels of 9,000 cd/m^2^ for the red-emitting, 14,700 cd/m^2^ for the green-emitting, and 700 cd/m^2^ for the blue-emitting unit. Again, these values are close to what is commonly shown for single emission unit OLEDs. The luminous efficacy as a function of the current density (*LE*-*j*) is shown in Fig. 3c. At a display-relevant brightness of 400 cd/m^2^, we obtain luminous efficacies of 13.9 lm/W, 29.2 lm/W, and 1.5 lm/W for the red, green, and blue emitting unit, respectively. Compared to the performance of single unit OLEDs, the power efficacy of our vertical pixel sub-units is thus not yet on par^9–11^. Clearly, the high degree of integration comes hand in hand with high complexity of the optical properties of such a multilayer system. To fully exploit the potential of this novel concept, further optical modeling is required, as well as exploration to what extend outcoupling concepts can be applied to this design^12^. The angular dependent emission spectra of each unit are shown in Fig. 4a–c. Despite the top-emission design and the application of three silver layers within the device, the obtained spectra only show minor micro-cavity features. The peak emission wavelengths of the red and green sub-units coincide with the peak photoluminescence (PL) wavelengths of the corresponding emitter species. For the blue sub-unit, the shoulder of the PL spectrum of 4P-NPD is predominantly outcoupled up to angles of around 30°. For higher angles, the blue emission spectra increasingly resemble the PL emission of 4P-NPD^13^.

The corresponding CIE color coordinates are shown as a function of the viewing angle in Fig. 4d–f. Due to the top-emission geometry with three semitransparent silver electrodes, one may expect the emission color to be angle-dependent^14–16^. However, apart from a small shift for the x- and y-coordinate of the green emission (Fig. 4e), the emission color of each unit is rather independent of the viewing angle which renders the vertically stacked devices very well suited for display applications.

**Figure 5:**
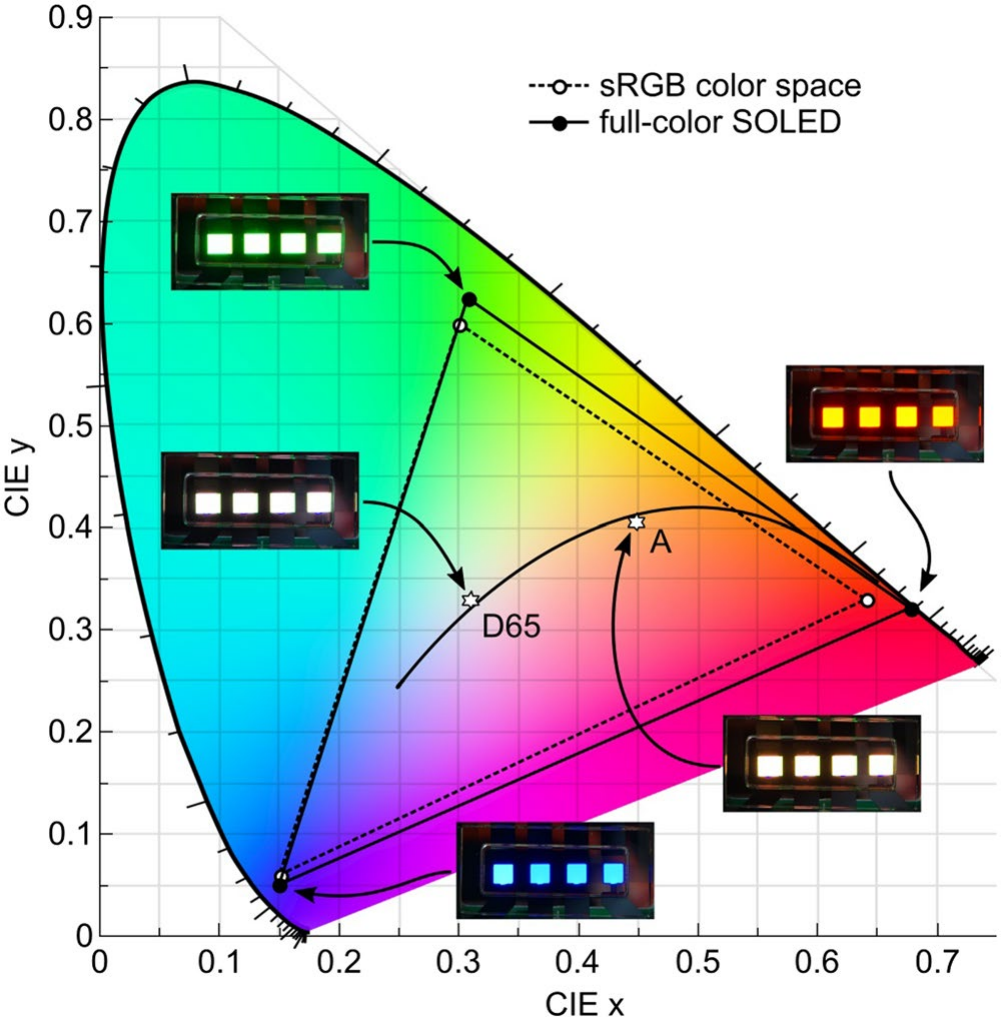
Chromaticity diagram showing the color coordinates of the pure emission from the red, green, and blue emission unit. The solid triangle spanned by connecting these three color points defines all colors that an RGB pixel can display by changing the contribution of the individual emitters. The dotted triangle represents the sRGB color space for comparison. The photographs show the device operating at the indicated color coordinates.

Figure 5 illustrates the color gamut provided by the different sub-units of our device for a 0° observation angle. For comparison, the sRGB color space is shown as a dotted line^3^. The pure emission color of the blue unit (0.15, 0.05) almost perfectly matches the blue primary (0.15, 0.06) of the sRGB color space, whereas the emission colors of the green (0.31, 0.62) and red unit (0.68, 0.32) are even superior to the respective green and red sRGB primaries, located at (0.30, 0.60) and (0.64, 0.33). The resulting color space is thus larger than sRGB, providing in particular more saturated colors in the green/yellow/red part of the CIE diagram. Apart from the emission of pure red, green, and blue light, the device can also display every color that can be represented as a superposition of the individual spectra of each unit (cf. Fig. 4a–d). Here, mixing of colors was achieved in a pulsed time-division multiplexing mode. All three subunits were driven at a fixed voltage, e.g. at 3.6 V. Within a time frame of 10 ms, each emission unit was switched on for a certain amount of time, corresponding to the desired spectral contribution (examplary timing diagrams are provided in Supplementary Fig. S3). This results in a refresh rate of 100 Hz which ensures that the human eye cannot resolve the sequentially emitted light pulses from the individual sub-units, but instead perceives a color equivalent to the integrated emission over several emission cycles^17^. For the presented pixel geometry (approx. 2.5 mm by 2.5 mm), we are able to drive the device with frequencies of up to 0.6–0.7 kHz with no or insignificant impact on brightness, efficiency and color quality. For higher frequencies, the RC time of the device (transport layer resistance times geometrical capacitance) becomes the limiting factor and luminance decreases. However, for smaller pixel size relevant for display applications, driving frequencies of several tens of kHz are possible.

**Figure 6:**
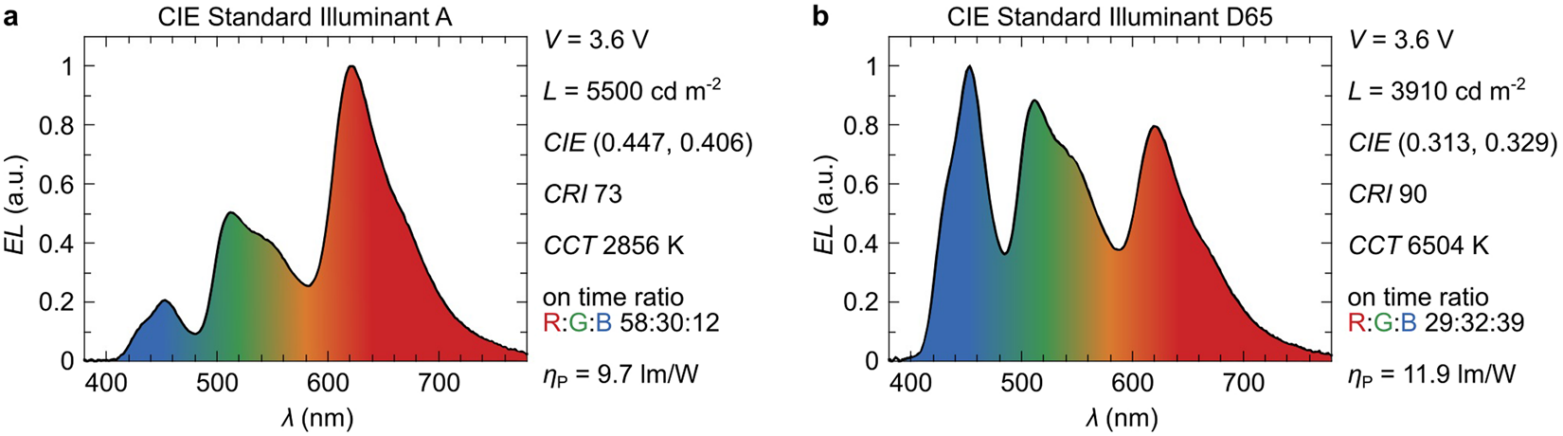
Emission spectra when the device is tuned to chromaticity coordinates representing the (**a**) CIE standard illuminant A and (**b**) CIE standard illuminant D65.

A wide range of color temperatures from 2700 K (incandescent bulb) to 6500 K (natural outdoor lighting) was available, making the presented approach ideal for customizable solid-state lighting (SSL) applications. The corresponding emission spectra for two driver configurations of the device are shown in Fig. 6. Matching the emission chromaticity coordinates to the CIE standard illuminant A was achieved by an R:G:B ratio of 58:30:12, which translates to pulse lengths of t_R_ = 5.8 ms, t_G_ = 3.0 ms, and t_B_ = 1.2 ms. Figure 6b shows a spectrum of the device emission when the color is tuned to match the chromaticity coordinates of CIE standard illuminant D65 (see Fig. 5 for CIE coordinates). The presence of emission from the red, green, and blue emitters within the device leads to a high color rendering index (CRI) of 90 when the emission color is tuned to match the daylight illuminant D65, and a CRI of 73 when tuned to warm-white color coordinates.

In addition to precisely adjustable white light, our approach is also able to produce a multitude of colors that can be used for high color gamut display applications, as well as ambient lighting, decorative elements, visual art, or novel product designs. A visual demonstration of this full-color SOLED concept is given in Supplementary Movie 1.

## Discussion

The demonstrated approach for an alternative pixel design to the conventional side-by-side fabrication of red, green, and blue subpixels in current display technology appears promising and excellent results were achieved in terms of electrical and optical performance. Still, the optical design of this multilayer SOLED concept bears substantial room for further improvement, e.g., by rearranging the positions of the red, green, and blue emitter within the device structure. In this work, we use a simple voltage-based driving scheme which is known to have drawbacks in terms of lifetime and image retention. A more preferable state-of-the-art driving scheme based on current instead of voltage can be applied to our design as well. Although the fabrication is challenging, it was shown that a stacking of three p-i-n-OLEDs is feasible. Despite the complex device architecture, an impressively high sample yield of almost 99% was achieved. The driving voltage for each individual unit is comparable to standard p-i-n single emission unit OLEDs; from an electrical point of view, the full-color SOLED device design is therefore well suited for integration into CMOS back-plane electronics where low pixel voltages are generally desired. However, the stacked architecture with its additional electrodes will require a modified CMOS back-plane layout, as the voltage/current is independently applied to three electrodes for each pixel. This ultimately results in more thin-film transistors, capacitors, and signal lines per pixel for the proposed SOLED design compared to a conventional active matrix OLED (AMOLED) display. The increased area requirement by the additional circuitry might lower the possible gain in pixel-per-inch, however, this sacrifice in pixel density can be kept to a minimum, using smart TFT/capacitor arrangements, as well as depositing the organic stack on top of the driving circuitry in combination with vertical interconnects (VIAs). This is necessary to establish a connection between the intermediate electrodes and the back-plane circuitry – our knowledge already a commonly used process for fabricating AMOLED displays in top-emission geometry.

Nowadays, pixel densities of more than 550 pixel per inch (ppi) can be achieved on an industrial production scale. Adapting the display fabrication process to the presented full-color SOLED pixel design, this value could easily be increased to 1400–1500 ppi — enough to allow for 4K-resolution on mobile phone-sized displays.

## Methods

Our device architecture comprises three p-i-n OLED units, emitting red, green, and blue light, stacked on top of each other, as schematically illustrated in Fig. 2. Internally, our devices contain four electrodes (labeled E1, E2, E3, and E4 in Fig. 2); however, E1 and E4 are electrically connected inside the device and thus are always at the same potential and can be addressed via a single external contact C1. E2 and E3 are independent counter-electrodes that can be accessed by external contacts C2 and C3. The polarity between C1, C2, and C3 controls whether the red-, green-, or the blue-emitting sub-unit of the device is active.

In order to realize efficient extraction of the light generated within each sub-unit, the different layer thicknesses in the device were optimized by optical simulation. For the presented device design, we were able to achieve a light outcoupling efficiency *η*_*out*_ of approximately 15% for each emission sub-unit. It is worth noting that a small fraction of efficiency in the range of 15–20% was intentionally sacrificed in return for a more stable emission color as a function of the viewing angle.

All layers were deposited in a UHV chamber at a base pressure of approximately 10^−8^ mbar onto cleaned glass substrates. The thickness and deposition rates of all layers were measured via quartz crystal monitoring (QCM). The evaporation rates varied from 0.3 Å/s for the emission layer to 1 Å/s for the p- and n-type layers. Doping was achieved by co-evaporation of the matrix material and the dopant.

The hole transport system in each unit was a combination of a p-doped, hole-conducting material (p-HTL) and a 10 nm layer of a non-doped, electron-blocking material (electron blocking layer, EBL). For the electron transport system, we used an n-doped, electron-conducting material (n-ETL) in combination with 10 nm of a non-doped, hole-blocking material (hole blocking layer, HBL). The blocking layer system separated the emission layer (EML) from the p-HTL/n-ETL, confining charge carriers and excitons to the respective EMLs. Using doped transport layers led to low driving voltages and allowed for modifications of the optical device properties without changing the electrical behavior.

As the p-HTL material in all three sub-units, we used 2,2′,7,7′-tetrakis-(N,N-di-methylphenylamino)-9,9′-spirobifluorene (Spiro-TTB) doped with 4 wt% 2,2′-(perfluoronaphthalene-2,6-diylidene)dimalononitrile (F6-TCNNQ). The n-ETL consist of 4,7-diphenyl-1,10-phenanthroline (BPhen) doped with cesium (Cs). The thickness of the p-HTL/n-ETL layer for the red-, green-, and blue-emitting sub-unit was 40/60 nm, 40/50 nm, and 20/60 nm, respectively. In the case of the red- and green-emitting sub-unit, aluminum(III) bis(2-methyl-8-quninolinato)-4-phenylphenolate (BAlq2) and 2,2′,7,7′-tetrakis-(N,N-diphenylamino)-9,9′-spirobifluorene (Spiro-TAD) were used as the HBL and EBL, respectively, whereas the HBL/EBL material combination for the blue sub-unit was BPhen/Spiro-TAD.

Deep-blue emission was obtained using 10 nm of the bulk emitter N,N′-di-1-naphthalenyl-N,N′-diphenyl-[1,1′:4′,1″:4″,1″′-quaterphenyl]-4,4″′-diamine (4P-NPD). The green EML was a double-emission structure, fabricated from a combination of 6 nm of the primarily hole-conducting 4,4′,4″-tris(N-carbazolyl)-triphenylamine (TCTA) matrix and 12 nm of the electron-conducting 2,2′,2″-(1,3,5-phenylen)tris(1-phenyl-1H-benzimidazol) (TPBi) matrix, both doped with 8 wt% of the phosphorescent green emitter tris(2-phenylpyridine)iridium(III) [Ir(ppy)3]. The red EML consisted of 15 nm N,N′-di(naphthalen-1-yl)-N,N′-diphenyl-benzidine (NPB) doped with 10 wt% of the red phosphorescent dye tris(1-phenylisoquinoline)iridium(III) [Ir(piq)3]. The reflective back electrode E1 (cf. Fig. 2) was a two-metal system, consisting of 40 nm silver (Ag) on top of 40 nm aluminum (Al). The highly transparent intermediate electrodes, as well as the top electrode, were a combination of 2 nm of gold (Au) and 12, 6, and 6 nm of silver (Ag) for electrodes E2, E3, and E4, respectively^5,6^. A 50 nm thick layer of NPB was used as capping layer on top of E4^18,19^. A detailed schematic of our device architecture is also provided in Supplementary Fig. 1. E1 and E4 were processed using the same shadow mask. As the organic layers do not completely cover E1, the two electrodes have a direct electrical connection. In this way, our device design allows for easy fabrication and the internal connection between E1 and E4 requires no additional space on the substrate. The mask set used for fabrication is shown in Supplementary Fig. S2. The active area of the device was 6 mm^2^. Prior to device investigation, the samples were encapsulated in a nitrogen glovebox. The overall sample yield was close to 99% (working subpixels: 426 out of a total of 432 tested [36 samples, 4 pixel per sample, 3 subpixel per pixel]).

The current-voltage-luminance (*j*-*V*-*L*) characteristics of our devices were measured using a source measure unit (SMU 2400, Keithley) in combination with a calibrated luminance meter (CS100A, Minolta). In order to provide correct values for the *EQE* and *LE* of each emission unit, angle-dependent measurements were performed in a spectro-goniometer setup that included a calibrated Ocean Optics USB4000 miniature spectrometer.

## Electronic supplementary material

Device operation of a full-color SOLED device

Supplementary Information
