# Supplementary material for: Three-terminal RGB full-color OLED pixels for ultrahigh density displays
Source: Sci Rep. 2018 Jun 26;8:9684. doi: 10.1038/s41598-018-27976-z (PMC6018739; doi:10.1038/s41598-018-27976-z)
Supplement: Supplementary file 2 — Supplementary Information [file 41598_2018_27976_MOESM2_ESM.pdf]

Supplementary Information

Three-terminal RGB full-color OLED pixels for ultrahigh density displays

Markus Fröbel[1], Felix Fries[1], Tobias Schwab[1], Simone Lenk[1], Karl Leo[1], Malte C. Gather[1,2] and Sebastian Reineke[1,*]

1. Dresden Integrated Center for Applied Physics and Photonic Materials (IAPP) and Institute for Applied Physics, Technische Universität Dresden, Nöthnitzer Str. 61, D-01187 Dresden, Germany

2. Organic Semiconductor Centre, SUPA, School of Physics and Astronomy, University of St Andrews, North Haugh, St Andrews KY16 9SS, UK

* Correspondence: Sebastian Reineke, Dresden Integrated Center for Applied Physics and Photonic Materials (IAPP) and Institute for Applied Physics, Technische Universität Dresden, Nöthnitzer Str. 61, D-01187 Dresden, Germany
E-mail: reineke@iapp.de
Tel +49-(0)351-463-38686
Fax +49-(0)351-463-37065

Detailed schematic of the device architecture and equivalent circuit

Figure S1 provides a more detailed schematic of the device architecture of a full-color SOLED pixel. It includes layer thicknesses, materials, doping concentrations, and electrical contacting.

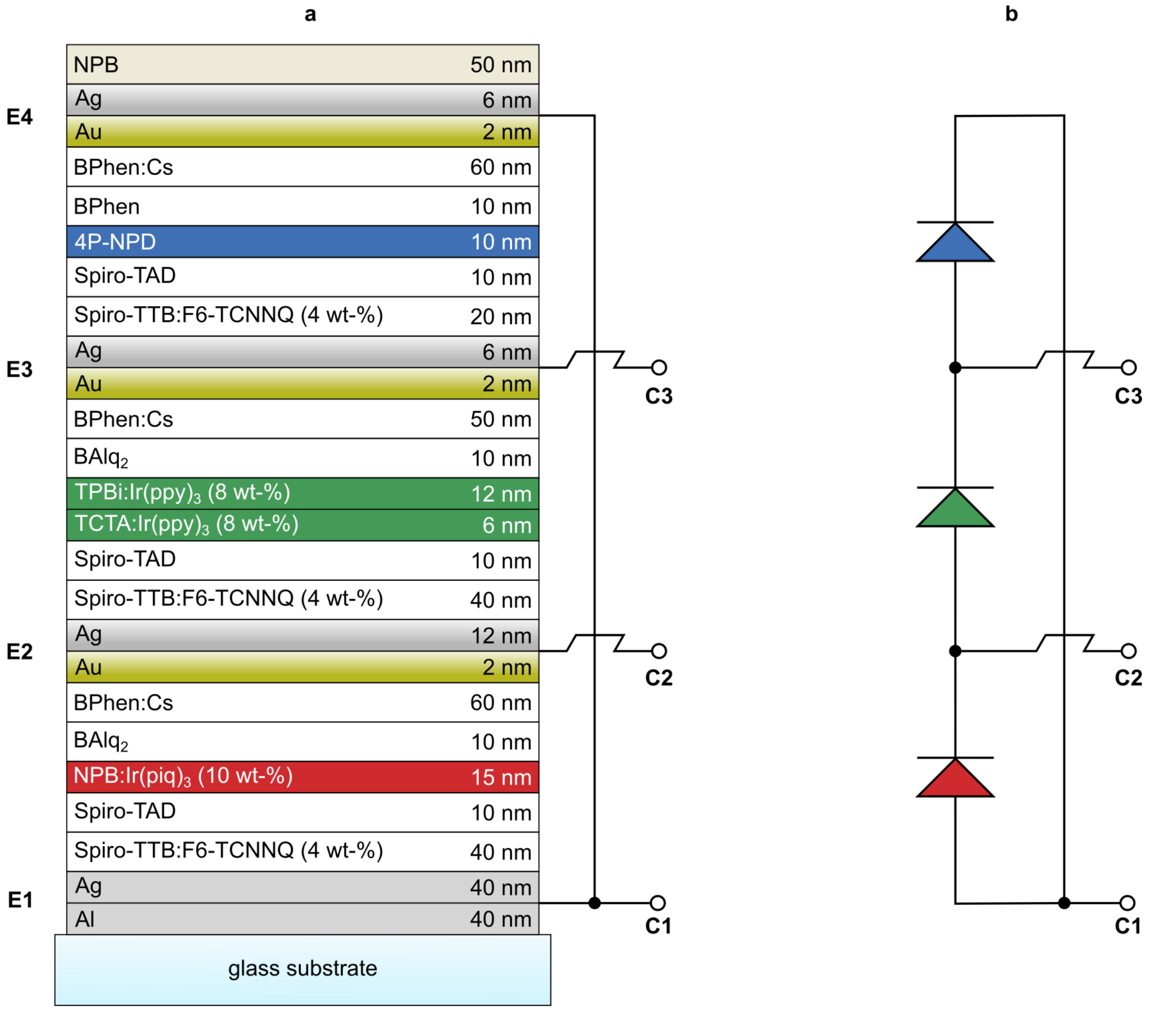

Figure S1 (a) Detailed schematic of the architecture of a full-color SOLED pixel. The device has a total of four electrodes (E1 to E4), however, as the bottom electrode (E1) and the top electrode (E4) are both connected to the same terminal C1, individual electrical driving of all three units can be accomplished by only three external terminals (C1, C2, and C3). (b) Equivalent circuit of a SOLED pixel.

Movies demonstrating device operation

Movie S1 Device operation of a full-color SOLED device. Pixels 1 – 3 (from left to right) alternately display the basic emission colors of their red (R), green (G), and blue (B) subpixels, whereas pixel 4 emits light that is a mixture of either R+G, R+B, or G+B.

Mask set overview

Figure S2 shows the four masks used for fabricating the presented SOLED device, as well as a stacked view of the mask overlap which defines the active pixel area.

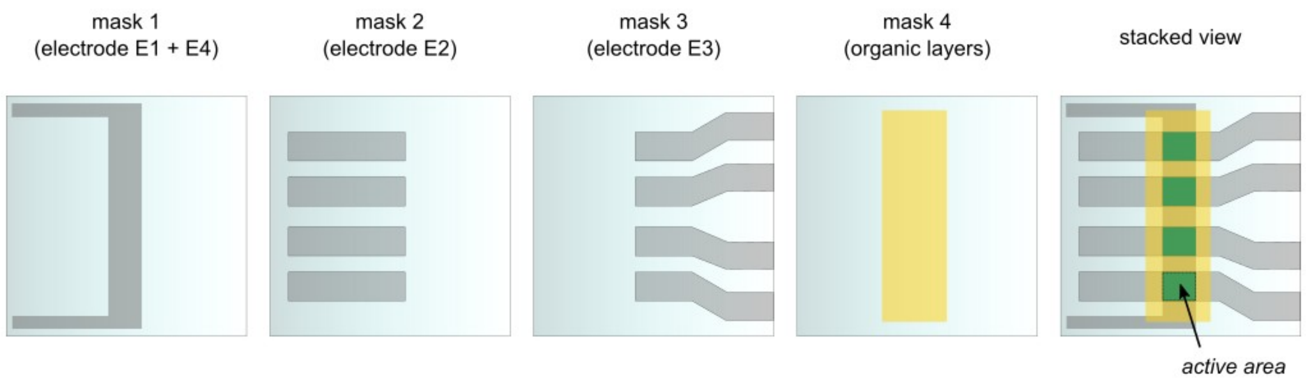

Figure S2 Mask set used for fabrication of the SOLED device. Overlapping mask areas define the active pixel region.

Timing diagrams (examples)

Figure S3 shows the corresponding timing diagrams for a selection of emission colors.

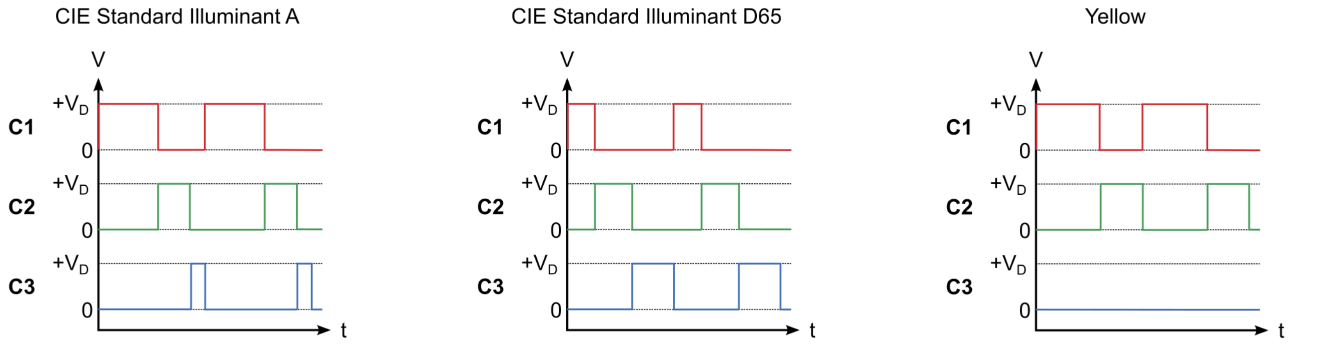

Figure S3 Examples for driving signals applied to the external terminals C1, C2, and C3 to achieve different emission colors.
